# The Effect of Deprotonation on the Reaction Kinetics of an Oxygen Scavenger Based on Gallic Acid

**DOI:** 10.3389/fchem.2019.00680

**Published:** 2019-11-07

**Authors:** Astrid F. Pant, Didem Özkasikci, Siegfried Fürtauer, Matthias Reinelt

**Affiliations:** ^1^Chair of Food Packaging Technology, TUM School of Life Sciences Weihenstephan, Technical University of Munich, Freising, Germany; ^2^Fraunhofer Institute for Process Engineering and Packaging IVV, Freising, Germany; ^3^Department of Food Engineering, Istanbul Technical University, Istanbul, Turkey

**Keywords:** active packaging, food packaging, oxygen absorber, polyphenol, 3,4,5-trihydroxybenzoic acid

## Abstract

Oxygen scavengers based on gallic acid (GA) usually contain a base that establishes the alkaline conditions necessary for the humidity-induced scavenger reaction. Here we measured the effect of sodium carbonate (Na_2_CO_3_) and sodium hydroxide (NaOH) on the reaction kinetics of such scavengers. The time-dependent oxygen absorption of aqueous GA solutions (pH 2.9–13.8) was determined and the results were described using a second-order kinetic model. We calculated the degree of deprotonation (DoD) of GA in the solutions from the species distribution curves, and assessed the effect of the DoD on the reaction kinetics. This revealed that both the reaction rate coefficient *k* and the scavenger capacity *n* were significantly affected by the DoD. If the DoD fell below 0.25, there was no significant reaction. Although *k* increased with the DoD, *n* reached a maximum at DoD = 0.6–0.7. In principle, target DoD values can be achieved using any base, but a strong base is more efficient because lower quantities are required. In our experiments, the amount of Na_2_CO_3_ required to reach the maximum DoD was more than twice that of NaOH. Our results provide the basis for the functional design of active packaging systems incorporating optimized GA-based oxygen scavengers.

## 1. Introduction

Gallic acid (GA), also known as 3,4,5-trihydroxybenzoic acid, is a natural polyphenol found in many plants either as a free acid or as a component of tannins (Shahidi and Naczk, 2003; Belitz and Grosch, 2013). When combined with a base, GA can be used as an oxygen (O_2_) scavenger for packaging applications, preventing the oxidation of sensitive food products (Langowski and Wanner, 2005). O_2_ scavengers based on GA (e.g., a mixture of GA and sodium carbonate) have a high O_2_ absorption capacity and can be integrated into packaging films (Ahn et al., 2016; Pant et al., 2017). Depending on the film structure, the scavenger can either improve the barrier function of the packaging material or absorb residual O_2_ from the headspace. The scavenger function is activated by humidity derived from the product or in the environment (Wanner, 2010).

**Figure 1:**
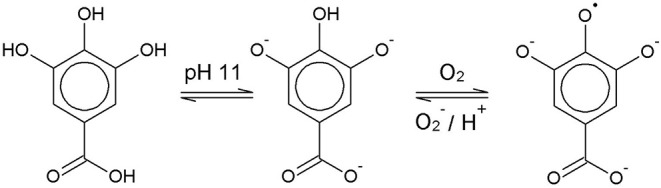
Formation of a gallate radical by single electron transfer at pH 11.

Chemically, the O_2_ scavenging function of GA-based O_2_ scavengers relies on the autoxidation of GA in alkaline solution. GA is a weak polyprotic acid (Figure 1) with four acidic protons that can be transferred to an acceptor base.

**Figure 2:**
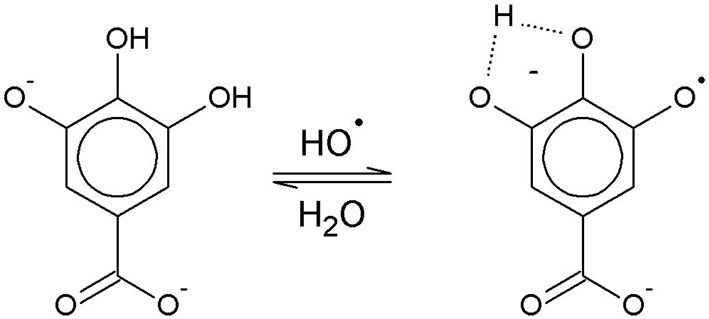
Hydrogen atom transfer from the gallate dianion to the hydroxyl radical at pH 9.

The solution pH determines the species distribution of GA, i.e., the proportions of GA and the different gallate anions present in the solution, and therefore also the type of GA radicals formed during autoxidation. For autoxidation at high pH Yoshioka et al. (2003) described radical formation by single electron transfer (SET) from the gallate anion to a dissolved O_2_ molecule (Figure 1). The by-product hydrogen peroxide (H_2_O_2_) undergoes homolysis to form two hydroxyl radicals when exposed to UV radiation from ambient sunlight (Holleman, 2007). Alternatively, metal ions such as Fe^2+^, which could be present as impurities, can be oxidized by H_2_O_2_ to form a hydroxyl radical and a hydroxyl anion (Walling, 1975). The hydroxyl radicals may in turn directly withdraw a hydrogen atom from GA in aqueous solution by hydrogen atom transfer (HAT) (Milenkovic et al., 2018). A HAT reaction at pH 9 is shown as an example in Figure 2.

**Figure 3:**
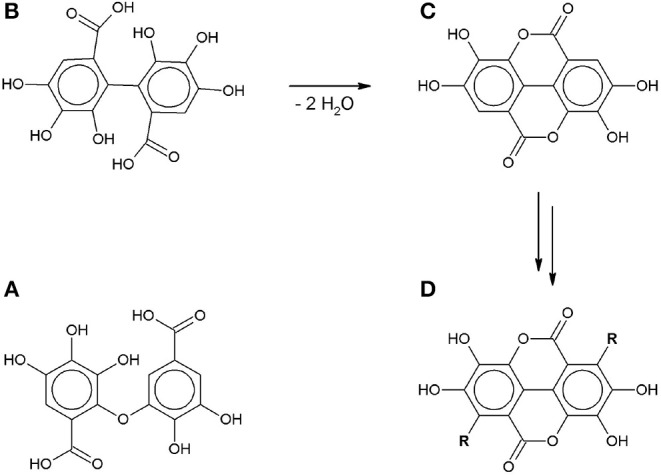
Gallic acid autoxidation intermediates: **(A)** C-O dimer (Oniki and Takahama, 2004). **(B)** C-C dimer (Caregnato et al., 2008). **(C)** Ellagic acid (Nikolic et al., 2011). **(D)** gallate-based polymer (R: gallate-derived group).

Both SET and HAT can lead to the formation of gallate radicals, which has been confirmed by electron spin resonance spectroscopy and shown in theoretical models (Yoshioka et al., 2003; Severino et al., 2009; Eslami et al., 2010). These gallate radicals can take part in further reactions. Tulyathan et al. (1989) proposed a multi-step oxidation mechanism including a ring-opening step, but did not report any qualitative or quantitative analysis of the reaction products. Hotta et al. (2001) proposed an oxidative polymerization mechanism in an alkaline environment in order to explain the unusually high number of transferred electrons compared to the number of hydroxyl groups. More recently some of the intermediates in this radical-induced polymerization pathway have been identified: C-O dimers (Oniki and Takahama, 2004), C-C dimers (Caregnato et al., 2008) and ellagic acid (Nikolic et al., 2011) (Figure 3).

An autoxidation that generates dark-colored reaction products has been described for several phenolic compounds (Cilliers and Singleton, 1989; Friedman and Juergens, 2000). Although the complete reaction pathway and the oxidation products have not yet been identified, the type and quantity of oxidation products changes with pH (Cilliers and Singleton, 1989; Friedman and Juergens, 2000). Wanner (2010) reported that a pH higher than 8 is necessary to enable the GA scavenger reaction and that the type of base used determined the reaction kinetics. Tulyathan et al. (1989) investigated the O_2_ absorption capacity of GA in the pH range 10–14 and observed a maximum first-order reaction rate constant at pH 12. For the polyphenol caffeic acid Cilliers and Singleton (1989) studied O_2_ absorption in the pH range 4–8 and reported increasing reaction rate constants at higher pH values.

Considering these results, the pH-dependent deprotonation of GA is likely to play a key role in selecting the autoxidation reaction and therefore the scavenger performance.

We previously used a 2:1 mass ratio GA:sodium carbonate as an O_2_ scavenger for packaging applications and investigated the effect of temperature and relative humidity on the second-order reaction rate constant and absorption capacity (Pant et al., 2018). However, it remains unclear which type of base and which mixing ratio achieves optimal oxygen scavenger activity.

Therefore, the aim of this work was to study the effect of the type of base on the oxygen scavenger kinetics of GA. The autoxidation of GA was studied in aqueous solutions adjusted to different pH values by adding either NaOH or Na_2_CO_3_. The second-order reaction rate coefficent and the absorption capacity were determined as well as their dependence on the deprotonation of GA.

## 2. Materials and Methods

### 2.1. Materials

Gallic acid monohydrate (GA, 99%) was obtained from ABCR (Karlsruhe, Germany). Water-free sodium carbonate (Na_2_CO_3_, 99.8%) and sodium hydroxide (NaOH, 98.8%) were obtained from Th.Geyer (Renningen, Germany).

To prepare aqueous GA solutions at different pH values, samples of 0.040 g GA powder and the appropriate amount of base were mixed with 5 mL destilled water in a Petri dish at 23 °C. Solutions were prepared with NaOH at pH 5.7, 7.9, 9.1, 11.1, 13.0, 13.8. Solutions were prepared with Na_2_CO_3_ at pH 5.1, 7.7, 8.3, 9.3, 10.4. The pH meter was calibrated with standard solutions at pH 7.0, 9.0, and 11.0. A GA solution without added base (pH = 2.9) was used as a reference.

### 2.2. Modeling the Acid-Base System

An aqueous solution of GA includes several acid-base equilibria, each characterized by its corresponding equilibrium constant *K*, as shown in Equations (1)–(5).

(1) $$\begin{array}{l} {\text{H}}_{4}\text{A}+{\text{H}}_{2}\text{O }⇄\;{\text{H}}_{3}{\text{A}}^{-}+{\text{H}}_{3}{\text{O}}^{+}{\;K}_{a1}=\frac{\left[{\text{H}}_{3}{\text{A}}^{-}\right]\left[{\text{H}}_{3}{\text{O}}^{+}\right]}{\left[{\text{H}}_{4}\text{A}\right]} \end{array}$$

(2) $$\begin{array}{l} {\text{H}}_{3}{\text{A}}^{-}+{\text{H}}_{2}\text{O }⇄\;{\text{H}}_{2}{\text{A}}^{2-}+{\text{H}}_{3}{\text{O}}^{+}K_{\;a2}=\frac{\left[{\text{H}}_{2}{\text{A}}^{2-}\right]\left[{\text{H}}_{3}{\text{O}}^{+}\right]}{\left[{\text{H}}_{3}{\text{A}}^{-}\right]} \end{array}$$

(3) $$\begin{array}{l} {\text{H}}_{2}{\text{A}}^{2-}+{\text{H}}_{2}\text{O }⇄\;{\text{HA}}^{3-}+{\text{H}}_{3}{\text{O}}^{+}{\;K}_{a3}=\frac{\left[{\text{HA}}^{3-}\right]\left[{\text{H}}_{3}{\text{O}}^{+}\right]}{\left[{\text{H}}_{2}{\text{A}}^{2-}\right]} \end{array}$$

(4) $$\begin{array}{l} {\text{HA}}^{3-}+{\text{H}}_{2}\text{O }⇄\;{\text{A}}^{4-}+{\text{H}}_{3}{\text{O}}^{+}{\;K}_{a4}=\frac{\left[{\text{A}}^{4-}\right]\left[{\text{H}}_{3}{\text{O}}^{+}\right]}{\left[{\text{HA}}^{3-}\right]} \end{array}$$

(5) $$\begin{array}{l} 2{\text{H}}_{2}\text{O }⇄\;{\text{H}}_{3}{\text{O}}^{+}+{\text{OH}}^{-}{\;K}_{w}=\left[{\text{H}}_{3}{\text{O}}^{+}\right]\left[{\text{OH}}^{-}\right] \end{array}$$

where H_4_A, ${\text{H}}_{3}{\text{A}}^{-}$, ${\text{H}}_{2}{\text{A}}^{2-}$, HA^3−^, and A^4−^ denote the GA species with 4, 3, 2, 1, and 0 acidic protons, respectively. Equations (1)–(4) describe the GA deprotonation equilibria characterized by the equilibrium constants pK_*a*_1__ = 4.39 which is related to the carboxylic proton and pK_*a*_2__ = 8.50, pK_*a*_3__ = 10.38, pK_*a*_4__ = 13 (Eslami et al., 2010; Jabbari, 2015), which are related to the phenolic hydroxy groups. Equation (5) shows the ion product of water, $K_{w}=10^{-14}$.

The acid-base system is further characterized by the mass balance of the GA species (Equation 6) and the charge balance (Equation 7):

(6) $$\begin{array}{l} {\text{c}}_{\text{A}}=\left[{\text{A}}^{4-}\right]+\left[{\text{HA}}^{3-}\right]\\ \;+\left[{\text{H}}_{2}{\text{A}}^{2-}\right]+\left[{\text{H}}_{3}{\text{A}}^{-}\right]+\left[{\text{H}}_{4}\text{A}\right] \end{array}$$

(7) $$\begin{array}{l} \left[{\text{H}}_{3}{\text{O}}^{+}\right]+\left[{\text{Na}}^{+}\right]=\left[{\text{OH}}^{-}\right]+\left[{\text{H}}_{3}{\text{A}}^{-}\right]+2\left[{\text{H}}_{2}{\text{A}}^{2-}\right]\\ \;+3\left[{\text{HA}}^{3-}\right]+4\left[{\text{A}}^{4-}\right] \end{array}$$

where c_A_ is the initial GA concentration and [A^4−^], [HA^3−^], $\left[{\text{H}}_{2}{\text{A}}^{2-}\right]$, $\left[{\text{H}}_{3}{\text{A}}^{-}\right]$, [H_4_A], [H_3_O^+^], [OH^−^], [Na^+^] are the equilibrium concentrations of the different GA species and the hydronium, hydroxide and sodium ions, respectively.

The system of Equations (1)–(7) was solved in MATLAB R2014a (The MathWorks, Inc., Natick, MA, USA) using the *fsolve* function. The pH (i.e., the concentration of ${\text{H}}_{3}{\text{O}}^{+}$), was varied systematically to obtain the pH-dependent distribution of the GA species. From this species distribution, the degree of deprotonation (DoD) was calculated according to Equation (8):

(8) $$\begin{array}{l} \text{DoD=}\frac{{\text{donated acidic H}}^{+}}{\text{total acidic }{\text{H}}^{+}} \end{array}$$

where total acidic H^+^ refers to the total amount of acidic H^+^ available from the GA molecules in the solution, and donated H^+^ refers to the total amount of acidic H^+^ transferred to the solution at a certain pH.

If Na_2_CO_3_, a biprotic base, is added to the solution, additional equilibria have to be considered as shown in Equations (9) and (10).

(9) $$\begin{array}{l} {\text{HCO}}_{3}^{-}+{\text{H}}_{2}\text{O }⇄\;{\text{CO}}_{3}^{2-}+{\text{H}}_{3}{\text{O}}^{+}{\text{ K}}_{\text{b}1}=\frac{\left[{\text{CO}}_{3}^{2-}\right]\left[{\text{H}}_{3}{\text{O}}^{+}\right]}{\left[{\text{HCO}}_{3}^{-}\right]} \end{array}$$

(10) $$\begin{array}{l} {\text{H}}_{2}{\text{CO}}_{3}^{*}+{\text{H}}_{2}\text{O }⇄\;{\text{HCO}}_{3}^{-}+{\text{H}}_{3}{\text{O}}^{+}{\text{ K}}_{\text{b}2}=\frac{\left[{\text{HCO}}_{3}^{-}\right]\left[{\text{H}}_{3}{\text{O}}^{+}\right]}{\left[{\text{H}}_{2}{\text{CO}}_{3}^{*}\right]} \end{array}$$

where ${\text{HCO}}_{3}^{-}$ and ${\text{CO}}_{3}^{2-}$ denote the bicarbonate and carbonate ions, respectively. ${\text{H}}_{2}{\text{CO}}_{3}^{*}$ accounts for both carbonic acid and dissolved CO_2_. The equilibrium constants we used were pK_*b*_1__ = 10.3 and pK_*b*_2__ = 6.3 (Lower, 1999).

### 2.3. Measurement of Oxygen Absorption

**Figure 4:**
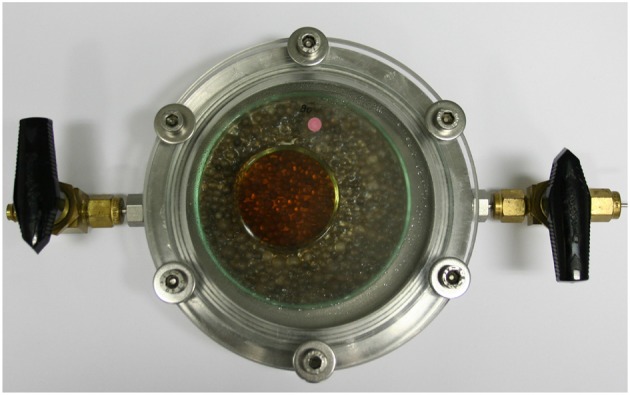
Experimental setup for oxygen absorption measurements.

The absorption of O_2_ by the aqueous GA solutions was measured in hermetically closed stainless-steel cells with a free headspace volume of 88 cm3 (Figure 4). The Petri dishes containing the GA solutions were placed in the cells and their O_2_ uptake from the headspace (initial gas atmosphere: air) was monitored for 30 days at 23°C using Fibox 4 Trace, a luminescence-based O_2_ detection system (PreSens Precision Sensing GmbH, Regensburg, Germany). To minimize the effect of O_2_ transport limits in the solution, the Petri dishes were only partially filled.

The *p*_O_2__ absorption data in hPa were converted to concentrations *c*_O_2__ using the ideal gas law (Equation 11).

(11) $$\begin{array}{l} c_{{\text{O}}_{2}}=n_{{\text{O}}_{2}}/V_{\text{HS}}=p_{{\text{O}}_{2}}/\left(R\cdot T\right) \end{array}$$

where *n*_O_2__ is the amount of substance of O_2_, *V*_HS_ is the headspace volume of the cell, *R* = 8.314 J mol^−1^ *K*^−1^ is the ideal gas constant, and *T* is the temperature in Kelvin.

Each experiment was carried out at least three times and the results are presented as the arithmetic mean with standard deviations.

The mean square error (MSE_exp_) of the observed O_2_ concentrations was calculated as shown in Equation (12):

(12) $$\begin{array}{l} {\text{MSE}}_{\text{exp}}=\sqrt{\frac{1}{N}\overset{m}{\underset{i=1}{\sum }}\overset{q}{\underset{j=1}{\sum }}{\left({\left[{\text{O}}_{2}\right]}_{\text{exp}}-{\left[{\text{O}}_{2}\right]}_{\text{mean}}\right)}^{2}} \end{array}$$

where *N* is the total number of observations, *m* is the number of parallel experiments, *q* the number of observations in one experiment, [O_2_]_exp_ the observed O_2_ concentration and [O_2_]_mean_ the arithmetic mean of all j^th^ observations of the *m* experiments.

### 2.4. Modeling the Oxidation Kinetics

The autoxidation of gallic acid can be described by the simplified net reaction shown in Equation (13) (Pant et al., 2018):

(13) $$\begin{array}{l} \text{GA}+{\text{nO}}_{2}\to {\text{GA}}_{\text{ox}} \end{array}$$

where GA represents all non-oxidized GA species, *n* is a stoichiometric factor and GA_ox_ accounts for the various reaction products. This simplified, application-oriented approach is based on the following assumptions: (i) we do not differentiate between the different GA species; (ii) the oxygen uptake is irreversible; and (iii) all oxidation products are summarized as GA_ox_.

The kinetic law of this reaction was approximated as a second-order elementary reaction and the reaction rate *r* was expressed as shown in Equation (14):

(14) $$\begin{array}{l} r=k\cdot \left[GA\right]\cdot \left[{\text{O}}_{2}\right] \end{array}$$

where [GA] and [O_2_] denote the concentrations of GA and O_2_, respectively [mol m^−3^] and *k* is the reaction rate coefficient [m^3^ mol^−1^ s^−1^]. Based on this kinetic law, the net consumption rates of GA and O_2_ were described with the system of ordinary differential equations (ODEs) shown as Equations (15) and (16)

(15) $$\begin{array}{l} \frac{d\left[\text{GA}\right]}{dt}=-k\cdot \left[\text{GA}\right]\cdot \left[{\text{O}}_{2}\right] \end{array}$$

(16) $$\begin{array}{l} \frac{d\left[{\text{O}}_{2}\right]}{dt}=-n\cdot k\cdot \left[\text{GA}\right]\cdot \left[{\text{O}}_{2}\right] \end{array}$$

The ODE system was solved in MATLAB R2014a using the multistep solver *ode15s*. The model was fitted to the experimental data to determine the kinetic parameters *k* and *n*. The fit was optimized using a downhill simplex method as described by Pant and Reinelt (2018).

## 3. Results and Discussion

### 3.1. Deprotonation of Gallic Acid in Aqueous Solution

**Figure 5:**
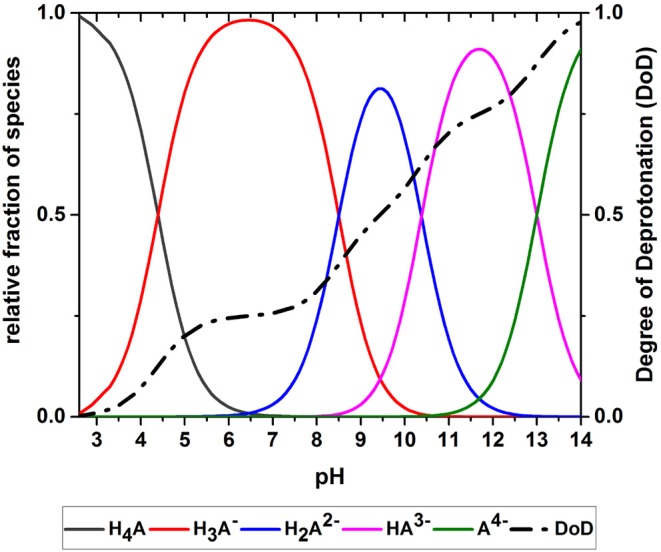
Species distribution of an aqueous gallic acid solution allowing the calculation of the degree of deprotonation (DoD).

The acid-base system studied in the oxidation experiments (i.e., an aqueous solution of GA) was described using Equations (1)–(10). GA is present as a range of deprotonated GA species that depend on the pH of the solution. The equilibrium concentrations of the different GA species at a given pH are determined by the acidity constants of the acidic protons. Figure 5 shows the simulated pH-dependent species distribution for aqueous GA solutions. When the pH is low, H_4_A is the dominant species, representing a GA molecule with four protons. As the pH increases, the relative proportions of the species change until almost all protons are donated at pH 14. The species distribution was used to calculate the DoD as a measure of the average deprotonation of GA (Figure 5). The DoD increases in line with higher pH values and almost complete deprotonation is achieved at pH~14. At pH 9.5 the DoD is 0.5, indicating that the GA molecules have donated an average of two acidic protons.

**Figure 6:**
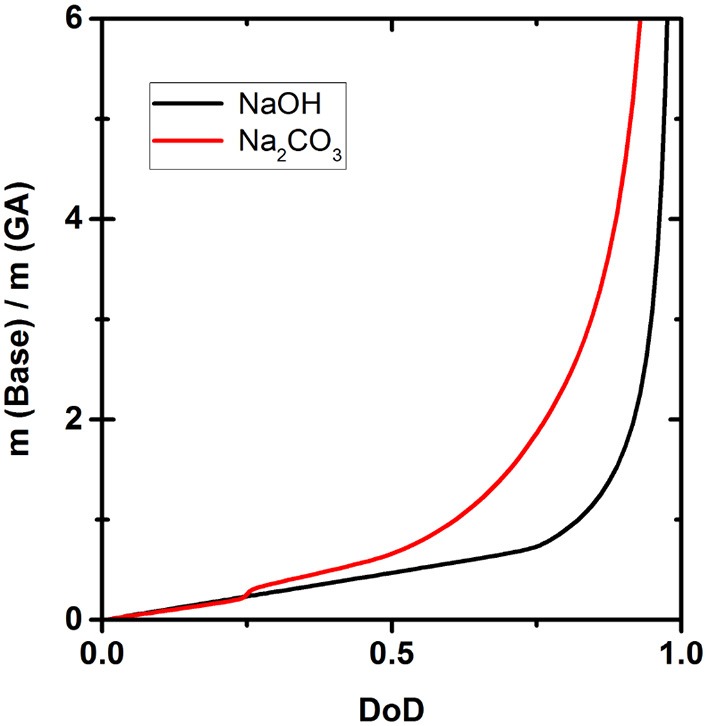
Effect of the base:gallic acid (GA) mass ratio on the degree of deprotonation (DoD).

Figure 6 shows how much NaOH or Na_2_CO_3_ is necessary to achieve a certain DoD according to the simulation. Due to the different strength of the two bases, the amounts differ significantly and the difference in the quantity of each base required becomes larger as the DoD increases. For example, to achieve a DoD of 0.75 using Na_2_CO_3_, base:GA weight ratio is 2, but this falls to only 0.8 when using NaOH.

### 3.2. Effect of pH on Oxygen Absorption

**Figure 7:**
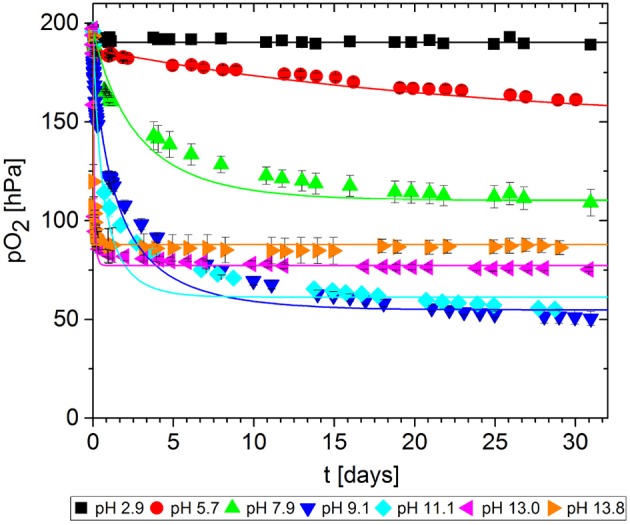
Oxygen absorption by aqueous solutions of gallic acid and NaOH (5 mL) at 23°C. Symbols shows the experimental data and lines show the simulation results.

**Figure 8:**
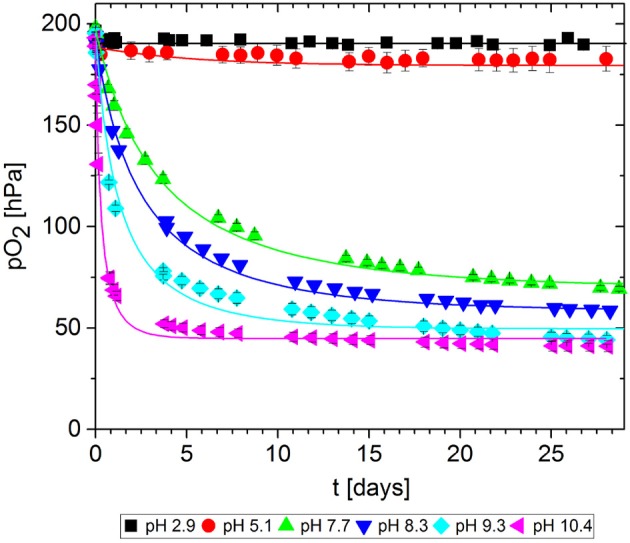
Oxygen absorption by aqueous solutions of gallic acid and Na_2_CO_3_ (5 mL) at 23°C. Symbols show the experimental data and lines show the simulation results.

In packaging materials, GA-based O_2_ scavengers can absorb O_2_ if water is present, i.e., in aqueous solutions comprising GA and a base. We assessed the effect of the base on the ability of GA to absorb O_2_ in solutions at different pH values, prepared by adding either NaOH (Figure 7) or Na_2_CO_3_ (Figure 8). Progress was monitored over 30 days of storage at 23°C.

The reference solution with no base added (pH = 2.9) did not absorb any O_2_. Regardless of which base was added, the more alkaline solutions generally absorbed greater quantities of O_2_ by promoting faster O_2_ uptake and thus more O_2_ absorption in total after 30 days. However, for NaOH solutions, the O_2_ absorption capacity peaked at ~10 and dropped again. Accordingly, the absorption capacity of GA at pH 11.1. was almost equal to that at pH 9.1, and the capacity was lower at pH 13.0 and 13.8. Tulyathan et al. (1989) also reported an O_2_ absorption peak but at slightly higher pH. In the case of Na_2_CO_3_, it was not possible to prepare solutions with a pH higher than 10.4 due to the buffer effect of the ${\text{HCO}}_{3}^{-}$ - ${\text{H}}_{2}{\text{CO}}_{3}^{*}$ system (pK_*b*_1__ = 10.3): The necessary quantities to achieve higher pH values would not dissolve in the GA solution.

**Figure 9:**
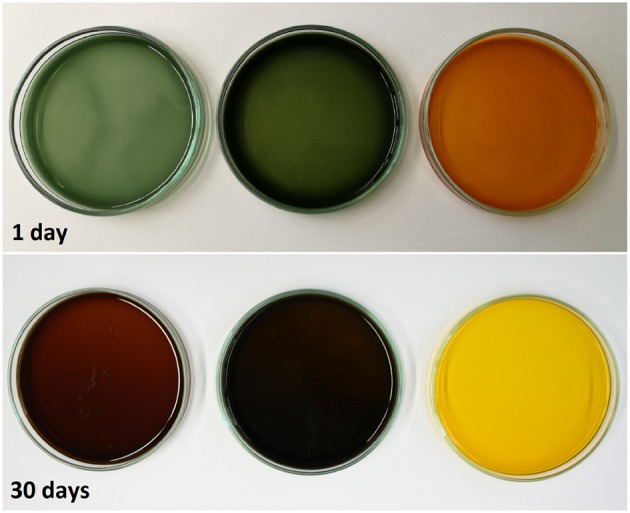
Color evolution of gallic acid solutions with pH 7.2, pH 11.1, and pH 13.0 (left to right).

The experiment was stopped after 30 days, but not all samples reached their maximum absorption capacity during this period because the scavenging reaction was too slow. The observed differences in O_2_ absorption must reflect a pH-dependent effect on the mechanism of the oxidation reaction, leading to different ways of incorporating O_2_ and thereby to different oxidation products. This was confirmed by the color development of the samples. Figure 9 shows samples from the solutions at pH 7.2, pH 11.1 and pH 13.0 after 1 day and after 30 days of storage. Initially (*t* = 0), the solutions were almost colorless with a slight yellow tint. Between days 1 and 30, the solutions at pH 7.2 and 11.1 changed from green to brown, whereas the solution at pH 13.0 change from organge to yellow. For autoxidation experiments in the pH range 2.6–11, Tóth et al. (2014) reported a similar profile of long-term color development. This qualitative observation supports previous reports that different polymerization products are formed during the autoxidation of GA in alkaline solutions (Cilliers and Singleton, 1989; Tulyathan et al., 1989; Hotta et al., 2001; Tóth et al., 2014). Further studies should be carried out to investigate the oxidation mechanism in more detail, including the effect of traces of metal ions on the reaction.

The O_2_ absorption measurements clearly demonstrated that pH has an effect on scavenger kinetics. However, the pH only provides information about conditions external to the reaction and, strictly speaking, the concept of pH can only be used to describe dilute aqueous solutions. For the design of O_2_ scavenger mixtures for packaging applications, it therefore seems more reasonable to describe the autoxidation process as a function of an intrinsic property of GA, the DoD.

The kinetic parameters *k* and *n* were determined for each experimental dataset by fitting a second-order kinetic model (Table 1). For most experiments, the simulation results were in good agreement with the empirical data: the root-mean-square error (RMSE) was in the same order of magnitude as the experimental error MSE_exp_. The fitted curves are shown in Figures 7, 8. These results show that the simple second-order model can be used to describe the O_2_ uptake by GA under diverse conditions.

**Table 1:** Model parameters.

**Base**	**pH**	**RMSE**	**MSE_**exp**_**	***k***	***n***
		**mol m^**−3**^**	**mol m^**−3**^**	**m^**3**^ mol^**−1**^ s^**−1**^**	
–	2.9	0.065	0.037	0^*^	0^*^
	5.7	0.139	0.063	6.292·10^−8^	0.68
	7.9	0.327	0.175	5.961·10^−7^	1.44
NaOH	9.1	0.320	0.084	1.169·10^−6^	2.16
	11.1	0.311	0.093	1.923·10^−6^	2.24
	13.0	0.146	0.058	2.995·10^−5^	2.01
	13.8	0.225	0.159	1.837·10^−4^	1.77
	5.1	0.065	0.056	2.871·10^−7^	0.15
	7.7	0.128	0.062	4.639·10^−7^	2.14
Na_2_CO_3_	8.3	0.113	0.035	6.625·10^−7^	2.25
	9.3	0.242	0.073	1.285·10^−6^	2.45
	10.4	0.158	0.098	5.868·10^−6^	2.50

* *No detectable reaction*.

**Figure 10:**
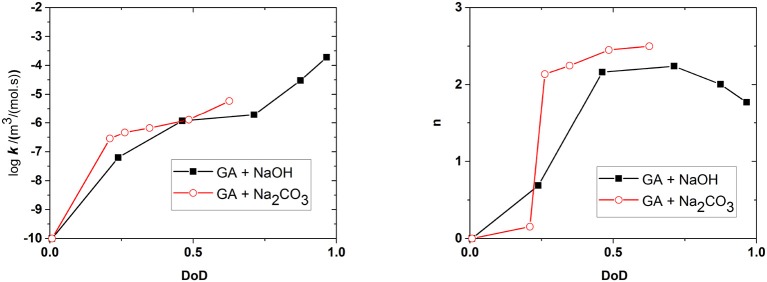
Effect of the degree of deprotonation (DoD) on the reaction rate coefficient *k* and absorption capacity *n*. Connecting lines are shown for clarity.

Figure 10 shows the effect of the DoD on the reaction rate constant *k* and the absorption capacity *n* in the presence of each base. We found that *k* increased in line with the DoD, but that *n* reached a maximum at a DoD value of 0.6–0.7 equating to a pH of 10–11 under our experimental conditions. When the DoD fell below 0.2–0.3 there was no significant O_2_ absorption. At a DoD value of 0.6–0.7 the predominant species in the solution are HA^3−^ and ${\text{H}}_{2}{\text{A}}^{2-}$ (Figure 5). These species can participate in an autoxidation reaction involving an SET step as described in Figure 1. Our results indicate that this pathway may be preferred in terms of achieving a maximum absorption capacity. In contrast, autoxidation based on A^4−^ is likely to involve a different reaction pathway leading to a lower absorption capacity.

Figure 6 shows the amounts of NaOH and Na_2_CO_3_ required to achieve a certain DoD in the solution and this relationship is the basis for the development of an optimized scavenger composition. For maximum O_2_ scavenger capacity, the DoD should be 0.6–0.7. This requires a base:GA mass ration of ~1.8:1 for Na_2_CO_3_ and ~0.8:1 for NaOH. As expected, a much smaller amount of the strong base NaOH is required to reach any given DoD compared to the weaker base Na_2_CO_3_. In terms of the design and processing of packaging materials incorporating scavengers based on GA, the amount of base required is important in terms of the precessability of the packaging material and the cost efficiency of the process.

## 4. Conclusion

In this study, we found that the amount and type of base added to an aqueous GA solution changes the O_2_ absorption properties of the system in terms of the reaction rate constant and also the maximum absorption capacity. The addition of a base changes the acid-base equilibrium of the solution and therefore the type and amount of GA species that are present, which in turn influences the autoxidation reaction and ultimately the absorption of O_2_. The key property that must be targeted when designing O_2_ scavengers based on GA is therefore the DoD. We found that no reaction occurred at DoD values below 0.25. The absorption capacity *n* reaches its maximum value when the DoD is 0.6–0.7 whereas the reaction rate constant *k* increases with the DoD. Accordingly, the optimum DoD can be achieved by adding either NaOH or Na_2_CO_3_, but a strong base is more efficient because a lower quantity is required to reach the desired DoD value. These results, together with the models describing the GA solution and the autoxidation reaction, provide guidance for the development of packaging systems incorporating O_2_ scavengers based on GA.

## Data Availability Statement

The datasets generated for this study are available on request to the corresponding author.

## Author Contributions

AP conceived and designed the experiments, performed some of the oxygen absorption measurements, conducted the simulations and parameter estimations, and wrote the paper. DÖ contributed to the experimental design and performed some of the oxygen absorption measurements. SF contributed to the analysis and interpretation of the results and helped to write and revise the paper. MR contributed to the experimental design as well as modeling and simulation techniques and revised the paper.

### Conflict of Interest

The authors declare that the research was conducted in the absence of any commercial or financial relationships that could be construed as a potential conflict of interest.
